# Supplementary material for: An ultrasensitive fiveplex activity assay for cellular kinases
Source: Sci Rep. 2019 Dec 19;9:19409. doi: 10.1038/s41598-019-55998-8 (PMC6923413; doi:10.1038/s41598-019-55998-8)
Supplement: Supplementary file 1 — Supplementary Information [file 41598_2019_55998_MOESM1_ESM.pdf]

SUPPLEMENTARY INFORMATION

An ultrasensitive fiveplex activity assay for cellular kinases

Christian M. Smolko[1] and Kevin A. Janes[1,2]

[1]Department of Biomedical Engineering, University of Virginia, Charlottesville, VA 22908
[2]Department of Biochemistry & Molecular Genetics, University of Virginia, Charlottesville, VA 22908

Correspondence and requests for materials should be addressed to K.A.J. (email: kjanes@virginia.edu)

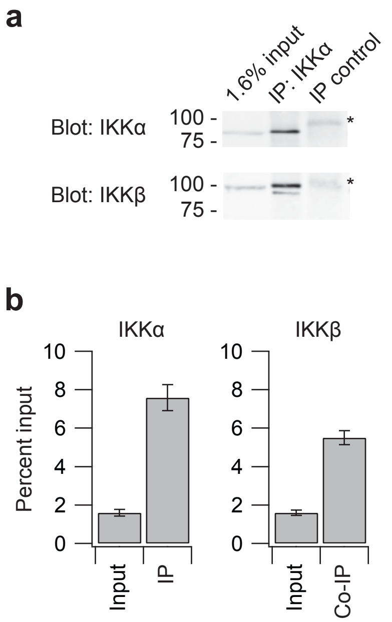

Supplementary Figure S1. Co-immunoprecipitation of IKKβ in a microplate-based immunoprecipitation of IKKα. **(a)** Immunoblot of IKKα and IKKβ after anti-IKKα immunoprecipitation of 500 μg lysate on Protein A/G microplates. Immunoprecipitation (IP) control uses an equivalent amount of naïve rabbit IgG. Asterisk indicates a nonspecific band from incompletely reduced IgG heavy chain. **(b)** Quantification of IKKα and IKKβ in IKKα immunoprecipitates relative to input. Data are shown as the mean ± s.e.m. of *n* = 4 independent immunoprecipitations. Uncropped immunoblots are available in Supplementary Fig. S3.

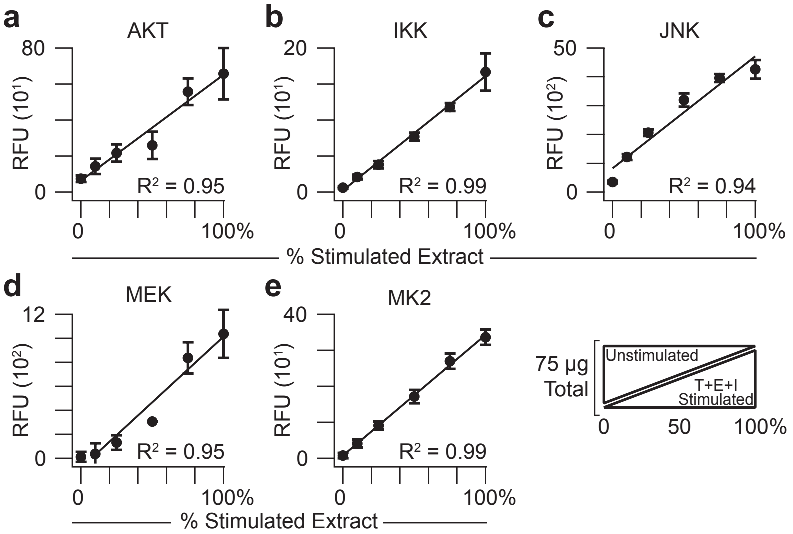

Supplementary Figure S2. Response characteristics of the fiveplex kinase assay with titrated extracts from AC16-CAR cells. Relative *in vitro* phosphorylation of substrates for **(a)** Akt, **(b)** IKK, **(c)** JNK, **(d)** MEK, and **(e)** MK2 was measured together as a function of proportionately stimulated cell extract added. Data are shown as the mean background-corrected relative fluorescence unit (RFU) ± s.d. of n = 4 assay replicates of 75 µg extract mixed in the indicated proportions of unstimulated AC16-CAR cells and cells treated with 20 ng/ml TNF, 100 ng/ml EGF, and 500 ng/ml insulin for 15 min (T+E+I stimulated).

a

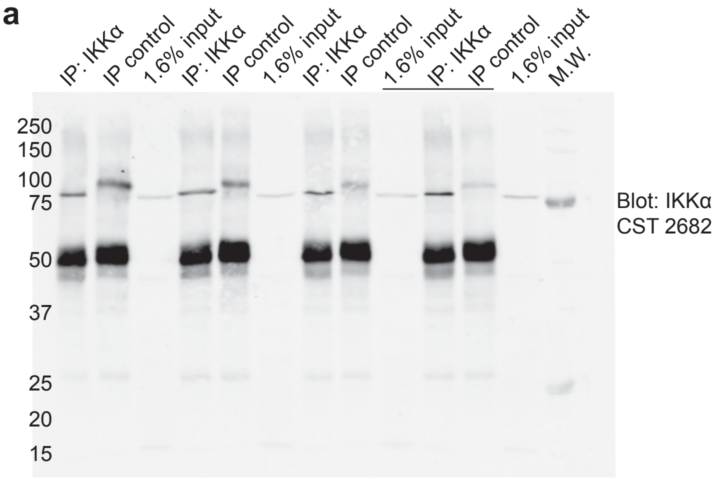

b

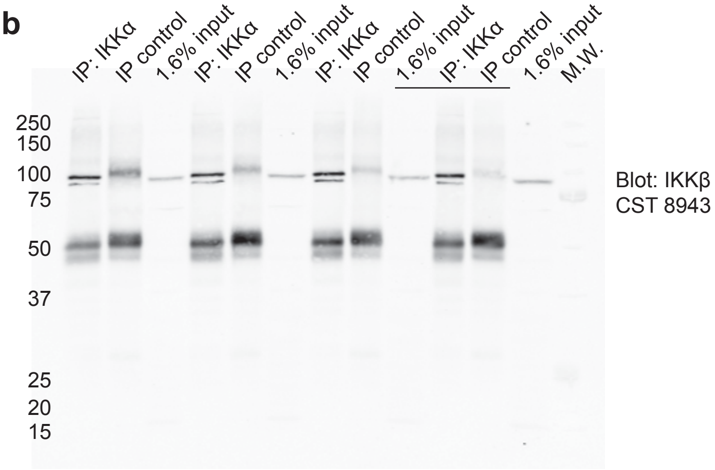

Supplementary Figure S3. Uncropped immunoblots for IKK immunoprecipitations in Supplementary Fig. S1. The exact lanes used in Supplementary Fig. S1a are underlined, and the results from four replicate immunoprecipitations are quantified in Supplementary Fig. S1b.

3x tag	Forward primer	Oligonucleotide template	Reverse primer
FLAG	gcgcagatctATGGATTACAAGGATGACG	ATGGATTACAAGGATGACGACGATAAGGATTACAAGGATGACGACGATAAGGATTACAAGGATGACGACGATAAG	gcgcgaattcggatccaagcttCTTATCGTCGTCATCCTTG
HA	gcgcagatctATGTACCCATACGATGTTCC	ATGTACCCATACGATGTTCCAGATTACGCTTACCCATACGATGTTCCAGATTACGCTTACCCATACGATGTTCCAGATTACGCT	gcgcgaattcggatccaagcttAGCGTAATCTGGAACATCG
Myc	gcgcagatctATGGAGCAGAAACTCATCTC	ATGGAGCAGAAACTCATCTCTGAAGAGGATCTGGAGCAGAAACTCATCTCTGAAGAGGATCTGGAGCAGAAACTCATCTCTGAAGAGGATCTG	gcgcgaattcggatccaagcttCAGATCCTCTTCAGAGATGAG
VSVG	gcgcagatctATGTACACCGATATAGAGATG	TACACCGATATAGAGATGAACAGGCTGGGAAAGTACACCGATATAGAGATGAACAGGCTGGGAAAGTACACCGATATAGAGATGAACAGGCTGGGAAAG	gcgcgaattcggatccaagcttCTTTCCCAGCCTGTTCATC
GluGlu	gcgcagatctATGGAGTATATGCCGATGGA	ATGGAGTATATGCCGATGGAGGAGTATATGCCGATGGAGGAGTATATGCCGATGGAG	gcgcgaattcggatccaagcttCTCCATCGGCATATACTCCT
AU1	gcgcagatctATGGACACCTACCGCTAC	ATGGACACCTACCGCTACATCGACACCTACCGCTACATCGACACCTACCGCTACATC	gcgcgaattcggatccaagcttGATGTAGCGGTAGGTGTCG

Supplementary Table S1. Oligonucleotides used for 3x epitope tag cloning. HindIII–BamHI–EcoRI restriction sites were added to the reverse primers for subsequent cloning of substrates.

Supplementary Note S1. Calculation of epitope-capture bead binding capacity at saturation.

The diameter of MagPlex microspheres is 5.6 μm, giving rise to $4\pi(5.6\ \mu m/2)^2 = 99\ \mu m^2$ of surface area per bead. The average diameter of an IgG molecule is ~30 nm[1], whose maximum projected area on the bead surface is approximated as $\pi(0.03\ \mu m/2)^2 = 0.0007\ \mu m^2$. Taking the ratio of these two areas yields (99 μm^2 per bead) / (0.0007 μm^2 per IgG) = ~140,000 IgG per bead. Each anti-epitope IgG molecule is bivalent, and each substrate is trivalent because of its 3x epitope tag (see Methods). We assumed each substrate was avidly bound to the bead at two sites; thus, ~140,000 substrate molecules could be bound to each bead at saturation. The terminated kinase reaction is incubated with ~2000 beads per barcode, giving (~140,000 substrate molecules per bead) x (~2000 beads per reaction) / (6.02 x 10^{23} molecules per mole) = ~0.47 femtomole per reaction.

Supplementary Note S2. Potential explanations for the higher variability of the Akt assay.

The repeatability of Akt activity measurements is consistently less precise than the other kinases in the assay (Fig. 2f), which we attribute to kinase phylogeny. Akt is a member of the AGC kinase family[2] and shares a substrate consensus with several other abundant kinases in the family, including RSK and S6K[3]. Selectivity of the immunoprecipitation is thus more important for Akt than for other kinases in the panel and may be difficult to achieve reliably with the five antibodies used in the assay. In addition, the GSK3α(1-97) substrate used for Akt is challenging, because the detected phosphoepitope encompasses two RxxS/T consensus sequences for other AGC kinases:

```
16 RARTSSFAE 24
   RxxT
     RxxS
```

The Thr19 site, if phosphorylated, would likely disrupt the epitope recognized by the anti-phospho-Ser21 antibody used for Luminex detection. The embedding of substrate consensus sequences, combined with the possibility of co-immunoprecipitating AGC kinases, would add technical uncertainty to Akt activity data.

SUPPLEMENTARY REFERENCES

1 Chen, Y., Cai, J., Xu, Q. & Chen, Z. W. Atomic force bio-analytics of polymerization and aggregation of phycoerythrin-conjugated immunoglobulin G molecules. *Mol. Immunol.* **41**, 1247-1252, doi:10.1016/j.molimm.2004.05.012 (2004).

2 Manning, G., Whyte, D. B., Martinez, R., Hunter, T. & Sudarsanam, S. The protein kinase complement of the human genome. *Science* **298**, 1912-1934 (2002).

3 Moritz, A. *et al.* Akt-RSK-S6 kinase signaling networks activated by oncogenic receptor tyrosine kinases. *Sci Signal* **3**, ra64, doi:10.1126/scisignal.2000998 (2010).
